# Intraoperative Polyuria as a Possible Complication of Anesthetic Agents and Spinal Procedures: A Case Series

**DOI:** 10.7759/cureus.88470

**Published:** 2025-07-21

**Authors:** Nikolas A Georgakis, Nicholas Goeman, Amit Aggarwal, Husong Li

**Affiliations:** 1 Department of Anesthesiology, University of Texas Medical Branch, Galveston, USA; 2 Department of Anesthesiology, Rocky Vista University College of Osteopathic Medicine, Englewood, USA

**Keywords:** arginine-vasopressin disorders, general anesthesia, intraoperative polyuria, ketamine, propofol, sevoflurane, spinal surgery, dexmedetomidine

## Abstract

Intraoperative polyuria, defined as urine output >2.5 mL/kg/hour during surgical procedures, can complicate fluid and electrolyte management. This series reviews cases of intraoperative polyuria in patients undergoing general anesthesia for spinal procedures. Urine output ranged from 2.69 to 3.69 mL/kg/hour during these procedures, which lasted from 8.5 to 12.5 hours. Dexmedetomidine, sevoflurane, ketamine, and/or propofol were used. Existing literature points to associations between these agents and transient arginine vasopressin (AVP) disorders that cause polyuria. All cases occurred in the setting of spinal procedures, which have also been associated with AVP disorders. The combination of multiple factors during surgical procedures that may increase the risk of AVP disorders has the potential to increase the incidence of intraoperative polyuria. None of the patients had a history of diabetes or AVP disorders, and no other causes of polyuria were identified. Awareness of the link between anesthetic medications, spinal procedures, and polyuria can improve the diagnosis and management of intraoperative polyuria.

## Introduction

Intraoperative polyuria - urine output >2.5 mL/kg/hour during a surgical procedure - can complicate fluid and electrolyte management, posing risks of dehydration, hemodynamic instability, and hypernatremia [1,2]. Intraoperative polyuria is commonly caused by iatrogenic overhydration, diuretic therapy, hyperglycemia, and arginine vasopressin (AVP) disorders [3,4].

AVP disorders are a potential cause of intraoperative polyuria. AVP is an endogenous hormone that allows the body to reabsorb free water from the collecting tubules of nephrons of the kidney, playing an important role in fluid and electrolyte balance in the body. AVP disorders are characterized by serum hyperosmolality (>300 mOsm/L), urine hypoosmolality (<300 mOsm/L), and polyuria (>2.5 mL/kg/hour) [4]. They can arise from central etiologies involving insufficient AVP secretion from the hypothalamic-pituitary axis, which is termed arginine vasopressin deficiency (AVP-D) [4]. Conversely, they can arise from nephrogenic etiologies involving renal resistance to AVP, which is termed arginine vasopressin resistance (AVP-R) [4]. To distinguish between the two, a desmopressin (DDAVP) trial can be performed. In AVP-D, DDAVP administration can lead to increased urine osmolality (up to 100% increase) [4]. In AVP-R, DDAVP administration will lead to minimal increases in urine osmolality [4]. Perioperative management in patients with AVP disorders requires vigilant monitoring and tailored fluid adjustments to prevent complications.

A possible cause of AVP disorders (both AVP-D and AVP-R) is the administration of certain anesthetic medications. In a literature review by Van Decar et al., the authors found several anesthetic medications that were linked to intraoperative polyuria [5]. These included dexmedetomidine, sevoflurane, ketamine, and propofol. Of these, dexmedetomidine has been most commonly associated with intraoperative polyuria, and propofol has been least commonly associated [5]. There are various proposed mechanisms for causing intraoperative polyuria for each of these agents, each related to either the release of AVP or the body’s response to AVP [5].

In addition, spinal surgery has been associated with polyuria, thought to be caused by AVP-D [6]. A proposed etiology is posterior pituitary circulatory disruption due to fat globules, thrombi, hypovolemia, or surgical traction [6,7]. This is thought to lead to hypoxia and tissue necrosis, preventing sufficient AVP release from the posterior pituitary gland [6]. It has been noted in many types of spinal procedures, including those involving the cervical, thoracolumbar, or lumbar spine [7]. The combined effects of certain anesthetic agents with the pathophysiologic impacts of spinal surgery may increase the risk of intraoperative polyuria [7].

It is important for anesthesiologists to use both clinical judgment and laboratory results to monitor for and diagnose intraoperative polyuria. Urine output should be closely monitored, as a urine output >2.5 mL/kg/hour is consistent with polyuria in adults [8]. Urine osmolality and specific gravity are also important indicators, with a urine osmolality of <300 mOsm/kg and a specific gravity of <1.003 being suggestive of polyuria [8]. In addition, plasma sodium >146 mmol/L and plasma osmolality >300 mOsm/kg can be clues to polyuria [8]. Although it occurs infrequently in the setting of intraoperative polyuria, hypernatremia is a concerning consequence of polyuria, as acute hypernatremia is associated with high morbidity and mortality due to cerebral cell shrinkage and its associated complications [9]. Once polyuria is identified, it is important to determine the cause and treat it accordingly. Correctable etiologies such as hyperglycemia, uremia, and diuretic administration should be ruled out [10].

The management of intraoperative polyuria includes multiple important modalities. First-line therapy involves replacing free water deficits with isotonic solution, as well as administration of DDAVP or vasopressin when indicated [5]. In addition, it is important to closely monitor the fluid and electrolyte status of the patient, as this can guide follow-up treatment [5].

This case series aims to guide the identification and evaluation of intraoperative polyuria, while also strengthening understanding of the perioperative care needs of patients with AVP disorders. Many stimuli that may occur intraoperatively have been associated with AVP disorders and, when experienced by a patient concomitantly, have the potential to increase the risk of intraoperative polyuria. By addressing these factors, this series seeks to contribute to enhanced clinical management and patient safety in surgical settings.

## Case presentation

A retrospective chart review was conducted on four patients who underwent general anesthesia at a tertiary care hospital between February 2024 and June 2024. The total intraoperative fluid administration in the cases ranged from 2.53 mL/kg/hour to 5.14 mL/kg/hour. Urine output ranged from 2.69 mL/kg/hour to 3.69 mL/kg/hour. Preoperative renal function was normal in all patients. Fluid administration was guided by blood pressure goals to maintain mean arterial pressures ≥65. Polyuria occurred during the middle to late stages of surgery. No DDAVP was administered in these cases. See Table 1 for the consolidated information.

**Table 1:** Intraoperative fluid intake and urine output with electrolyte values

Case	Patient weight (kg)	Fluid intake (mL/hr)	Urine output (mL/hr)	Fluid intake by weight (mL/kg/hr)	Urine output by weight (mL/kg/hr)	Estimated blood loss (mL)	Intraoperative sodium (mmol/L)	Intraoperative glucose (mg/dL)
1	103.7	379.61	316	3.66	3.05	300	138-140	118-142
2	119.7	434.16	442	3.62	3.69	250	137-139	107-129
3	68	171.97	222	2.53	3.26	200	135-136	92-103
4	65.4	336.46	176	5.14	2.69	450	136 (venous)	125 (venous)

All of these cases of intraoperative polyuria occurred in the setting of a spinal procedure. All patients had invasive blood pressure monitoring through an arterial line, which was used to guide fluid management. One of the procedures was a lumbar laminectomy, foraminotomy, and facetectomy; two were posterior lumbar interbody spinal fusions; and one was a cervical laminectomy with implant. The patients’ ages at the time of the procedure ranged from 15 to 75 years old. All patients had a past medical history of spinal pathology, and some had additional past medical history such as coronary, respiratory, and/or endocrine disease. See Table 2 for the consolidated information.

**Table 2:** Patient and procedure information CAD: coronary artery disease; GERD: gastroesophageal reflux disease; HTN: hypertension; MI: myocardial infarction; OSA: obstructive sleep apnea

Case (age)	Past medical history	Spinal location	Procedure	Complications
1 (75)	CAD, MI, HTN, OSA, GERD, lumbar stenosis	Lumbar	Laminectomy, foraminotomy, and facetectomy	Incidental durotomy of the right L5 nerve root at the shoulder and the root sleeve with evidence of cerebrospinal fluid leak
2 (44)	OSA, lumbar stenosis, lumbosacral degenerative disc disease	Lumbar	Posterior lumbar interbody spinal fusion	None reported
3 (54)	Hyperparathyroidism, scoliosis, cervical myelopathy, cervical radiculopathy	Cervical	Laminectomy with implant	None reported
4 (15)	Lumbosacral anterolisthesis, lumbosacral spondylolysis	Lumbar	Posterior lumbar interbody spinal fusion	None reported

In case 1, 0.8-0.9 MAC sevoflurane was used as the maintenance anesthetic, with ketamine infused throughout the case. In case 2, 0.5 MAC sevoflurane was used, with propofol and remifentanil infused throughout the case. In case 3, total intravenous anesthesia was done with propofol and remifentanil throughout. In case 4, 0.3-0.4% isoflurane was used, with propofol infusion throughout the case and ketamine infusion for slightly less than the first half of the case, as the patient was given IV methadone for analgesia during the second half of the case. Ketamine was administered intraoperatively in three of the four cases (1, 3, and 4). Dexmedetomidine was given in case 4 throughout the case. See Table 3 for the consolidated information.

**Table 3:** Intraoperative anesthetic agents MAC: minimal alveolar concentration; TIVA: total intravenous anesthesia, mcg: micrograms, kg: kilograms, min: minutes

Case (total body weight in kg)	Volatile agent maintenance amount	Propofol infusion rate and total	Remifentanil infusion rate and total	Ketamine infusion rate and total	Dexmedetomidine infusion rate
1 (103.7)	Sevoflurane (0.8-0.9 MAC)	-	-	Infusion rate: 3 mcg/kg/min Total: 200 mg	-
2 (119.7)	Sevoflurane (0.5 MAC)	Infusion rate: 75-90 mcg/kg/min Total: 5350.92 mg	Infusion rate: 0.07-0.1 mcg/kg/min ‘ Total: 4267 mcg	-	-
3 (68)	None (TIVA)	Infusion rate: 120-150 mcg/kg/min Total: 4999.65 mg	Infusion rate: 0.05-0.08 mcg/kg/min Total: 3000 mcg	50 mg bolus during hour 9 of the case	-
4 (65.4)	Isoflurane (0.3-0.4%)	Infusion rate: 90-150 mcg/kg/min Total: 5500.8 mg	-	Infusion rate: 3 mcg/kg/min Total: 50 mg	Infusion rate: 0.3-0.5 mcg/kg/hr Total: 82.36 mcg

## Discussion

These cases have been reviewed in the context of different causes of intraoperative polyuria. None of the patients in this study had a documented history of AVP disorders. Anesthetic agents used in these cases have been associated with drug-induced AVP disorders [5]. These include dexmedetomidine, sevoflurane, ketamine, and propofol. Different mechanisms causing AVP disorders have been proposed for these agents. Dexmedetomidine has been shown in canine and rat studies to decrease both the release of and response to AVP, indicating mixed AVP-D and AVP-R mechanisms, although human studies have not yet been conducted [5]. It is also thought that dexmedetomidine acts on imidazoline receptors via a non-AVP-dependent mechanism to increase sodium and free water excretion [11]. Sevoflurane has been proposed to interfere with the aquaporin 2 signaling pathway, rendering the action of AVP on nephron collecting tubules less effective [12]. This effect is thought to be transient [12]. Ketamine has been associated with AVP-D, likely through its inhibition of N-methyl-d-aspartate receptors, leading to the decreased ability of glutamate to stimulate AVP release [13]. Propofol is thought to increase the effects of GABA inhibition of AVP release, specifically through binding the GABAA receptor, causing an AVP-D effect [14]. Considering that at least two out of these four implicated agents were used in each case, it is possible that they contributed to the polyuria experienced by these patients.

**Table 4:** Mechanisms of polyuria due to anesthetic medications AVP: arginine vasopressin, AVP-D: arginine vasopressin deficiency, AVP-R: arginine vasopressin resistance

Medication	Mechanisms
Dexmedetomidine	Combined AVP-D and AVP-R + AVP-independent sodium and water retention
Sevoflurane	Aquaporin 2 signaling disruption (AVP-R)
Ketamine	Inhibition of NMDA receptors (AVP-D)
Propofol	GABA-mediated inhibition of AVP release (AVP-D)

In addition, each of the four patients in this case series underwent spinal surgery, which has been associated with intraoperative polyuria [6,7]. Intraoperative polyuria has been shown to occur during various types of spinal procedures [7]. Cases of spinal-surgery-associated polyuria are thought to be caused by AVP-D due to hypoperfusion of the posterior pituitary gland [6]. The mechanisms for this may include disruption of posterior pituitary circulation by fat globules, thrombi, hypovolemia, or surgical traction [6,7]. Anesthetic agents have been associated with increased risk of intraoperative polyuria during spinal procedures, which may underscore additive polyuric effects of certain agents with the pathophysiologic impacts of spinal surgeries [7].

Moving forward, it is important to keep in mind that anesthetic agents synergistically, especially in the setting of spinal procedures, may have the potential to cause intraoperative polyuria. These include dexmedetomidine, sevoflurane, ketamine, and propofol. Prompt diagnosis is crucial, which can be achieved through close monitoring of urine output, urine osmolality and specific gravity, and measurement of other serum values such as glucose and urea [6]. In conjunction with fluid management, possible administration of DDAVP in cases of hypernatremia, and fluid/electrolyte monitoring, the potential offending agent should be discontinued when reasonable [5]. These management strategies are likely to lead to cessation of polyuria in cases caused by anesthetic agents, as it is thought that the polyuria caused by these agents is reversible and transient [5].

Other causes of intraoperative polyuria include hyperglycemia, iatrogenic agents such as diuretics, and overhydration. In these cases, intraoperative hyperglycemia was not observed, and diuretic agents were not administered. In addition, conventional perioperative fluid management recommendations discuss administering 3-5 mL/kg/hour of fluids [15]. This amount was not exceeded, except in case 4, where the patient received 5.14 mL/kg/hour. Fluid administration was guided by intraoperative hemodynamic monitoring and blood pressure goals. Therefore, etiologies of AVP disorders should be investigated as the potential cause of intraoperative polyuria in these cases. Another agent used in all four cases was tranexamic acid, which was given as 1-2 gram boluses. It is an antifibrinolytic agent commonly used to reduce perioperative bleeding by inhibiting the breakdown of clots [16]. While the agent is not commonly associated with polyuria, the pharmacology behind it can induce microthrombi, leading to cortical necrosis and subsequent acute kidney injury at high doses [16]. In patients with renal impairment, the clearance can be reduced, leading to renal dysfunction and possible oliguria instead of polyuria [16].

This study has limitations. Due to its retrospective nature, there was limited control over confounding variables. These variables include choices of anesthetic medications and their doses, as well as the type and quantity of intravenous fluids. Furthermore, measurement of values such as urine osmolality and urine specific gravity perioperatively would have helped clarify the cause of intraoperative polyuria in these cases. This limits the ability to diagnose AVP disorders as the cause of polyuria with certainty. Additionally, by nature of being a case series, this study has a small sample size. Future prospective studies could benefit this area of research and further elucidate the potential link between these anesthetic medications and intraoperative polyuria. More information is needed to contribute to understanding of the clinical implications of these findings.

## Conclusions

Certain anesthetic agents, such as dexmedetomidine, sevoflurane, ketamine, and propofol, may increase the incidence of intraoperative polyuria. This may be particularly relevant when these agents are used in conjunction and during spinal procedures. The polyuric effects of these medications and spinal procedures are thought to be due to mechanisms related to AVP disorders. Through awareness of these factors, in addition to meticulous monitoring, anesthesiologists can diagnose and manage intraoperative polyuria. Specifically, anesthesiologists should quantify urine output, assess serum sodium and osmolality, measure urine osmolality and specific gravity, and closely monitor hemodynamics and electrolyte changes. Prospective studies are needed to confirm these findings and further evaluate the link between anesthetic agents, spinal procedures, and intraoperative polyuria.
